# Nebraska

**Published:** 1899-04

**Authors:** A. T. Peters

**Affiliations:** Secretary


Secretary.

### NEBRASKA VETERINARY MEDICAL ASSOCIATION.

THE semi-annual meeting was held at the Capital Hotel, Lincoln, February 21, 1899. It was one of the most interesting sessions the Association has ever held, as evidenced by the intense interest in the discussions following the papers. The meeting was called to order at 3 o'clock by Vice-President Dr. George P. Tucker, of Lincoln. The following members were present: Drs. E. T. Bowers, Hastings; A. Bostrom, Minden; J. J. Drasky, Crete; C. F. Leslie, Wahoo; A. T. Peters, Lincoln; H. L. Ramacciotti, Omaha; G. P. Tucker, Lincoln; Dr. V. Schaefer, Tekamah; S. E. Cosford, Lincoln; G. R. Young, Omaha; J. S. Anderson, Seward; and A. W. Thomas, Lincoln. J. D. Sprague, David City; Mr. Heath, of the Nebraska *Farmer;* Mr. Fassett, of the *Western Swine Breeder*, and V. C. Barber, of the Agricultural Experiment Station, were present as visitors.

It was decided to hold the next meeting in connection with the Iowa Veterinary Association at Omaha next fall. An invitation was extended to the Missouri Valley Veterinary Association to meet at that session.

House roll 475, the new veterinary bill, demanded much attention, and important testimony was brought out relative to its necessity for the protection of the veterinary profession, as well as the livestock interests of the State. Many outbreaks of rabies and other contagious and infectious diseases were reported, showing the necessity of Nebraska having a State veterinarian. It was noted that Nebraska stands alone as the only State without such an officer. The bill has already passed the Livestock and Grazing Committee, and indications were reported as favorable for its passage.

Resolutions were passed extending the sympathies of the Association to Dr. Solomon Bock, of Denver, who was recently stricken with paralysis and has been obliged to abandon an extensive practice. Resolutions were also passed expressing the Association's thanks to Dr. Gresswell, of the same city, for his excellent work at the National Stock Growers' Association in the interest of sanitary science and the veterinary profession. On account of the recent action of the trustees of the Ames Veterinary College, of Iowa, relative to the removal of a competent veterinarian from their faculty, the Secretary was instructed to inquire into the matter, to ascertain the standard of the institution.

V. C. Barber, assistant animal pathologist of the Agriculture Experiment Station of the University, was elected honorary member of the Association. Officers continued for the ensuing term are: Dr. V. Schaeffer, Tekamah, President; Dr. George P. Tucker, Lincoln, Vice-President; Dr. A. T. Peters, Lincoln, Secretary; Dr. J. S. Anderson, Seward, Treasurer.

After adjournment of the afternoon session the members, upon invitation from Mrs. A. T. Peters, attended a delightful luncheon at her home.

In the evening Dr. J. S. Anderson, of Seward, read a paper on "Fistulous Withers and How to Operate Them." He regarded cutting as the surest method of effecting a cure. Though this method met with some opposition in the discussion that followed, Dr. Anderson very ably defended his manner of treatment of this very common affection with which the veterinarian has to cope.

Dr. J. J. Drasky, of Crete, then read a paper entitled "What I Saw at Omaha." After paying a tribute to the Association for the able manner in which they entertained the national meeting of the American veterinarians at Omaha last September, he touched upon the benefits to be derived from attending these conventions. The main object of his paper, however, was to reprimand a veterinarian, who, in the course of a clinic before the above-mentioned national association, acted in a most unprofessional manner and took occasion to belittle a skilled Omaha veterinarian. It was Dr. Drasky's desire that this reprimand be not made public; but the members, seeing the opportunity of thrusting a blow at "quacks," unanimously decided to publish his paper in the leading veterinary journals of the country.

"Cornstalk Disease in Cattle and Horses" was the subject of a most excellent paper by Dr. A. Bostrom, of Minden. He regarded death in cornstalk fields as not due always to the stalks, but that "fungus disease" was the cause of much of the trouble. The discussion was long and earnest, and many new facts were brought to light that may be fruitful of much interesting knowledge in the near future.

"My Experience with Black-leg Vaccine" was read by Dr. Peters, the author of the paper, Dr. M. V. Byers, of Osceola, being absent. Dr. Byers stated in a clear and concise manner his extensive experience with the method of preventing the disease, and showed plainly that it was the surest and safest method of prevention. Dr. A. T. Peters, of the University, then followed with a history of black-leg vaccination, tracing it from its introduction in the old country up to the present time. He explained how the vaccine was prepared and used, stating that it could not be obtained free of charge from the Agricultural Department. He said that 33,000 cattle had been vaccinated in Nebraska during the past year with most gratifying results. Dr. Peters' talk ended the programme, which, on account of the long discussions, lasted until after midnight.

The Association voted to attend in a body the Improved Stock Breeders' and Swine Breeders' meeting.

A. T. PETERS,
Secretary.

